# Unexpected aspects in the dynamics of horizontal gene transfer of prokaryotes: the impact of outer membrane vesicles

**DOI:** 10.1007/s10354-018-0642-2

**Published:** 2018-08-06

**Authors:** Branko Velimirov, Carmen Ranftler

**Affiliations:** 1 Center for Pathobiochemistry and Genetics, Medical University of Vienna, Währingerstr. 10, 1090 Vienna, Austria; 2 Center of Anatomy and Cell Biology, Medical University of Vienna, Schwarzspanierstr. 5, 1090 Vienna, Austria

**Keywords:** Serial transduction, Sequence analysis, *E. Coli* AB1157, *Ahrensia kielensis*, *Pseudoalteromonas marina*, Serielle Transduktion, Sequenzanalyse, *E. coli* AB1157, *Ahrensia kielensis*, *Pseudoalteromonas marina*

## Abstract

Horizontal gene transfer (HGT) was observed by incubation of an amino acid-deficient strain of *Escherichia coli* (AB1157) with particles gained from an oligotrophic environment, when all deficiencies were restored with frequencies up to 1.94 × 10^−5^ and no preference for a single marker. Hence, the DNA transfer to the revertant cells was carried out by generalized transduction. Those particles display structural features of outer membrane vesicles (OMVs) but contain high amounts of DNA. Due to a process called serial transduction, the revertant’s particles were likewise transferring genetic information to deficient *E. coli* AB1157 cells. These results indicate a new way of HGT, in which mobilized DNA is transferred in particles from the donor to the recipient. Extracted OMV-associated DNA of known alpha-, and gamma-proteobacterials, *Ahrensia kielensis* and *Pseudoalteromonas marina*, respectively, was larger than 30 kbp with all sequences in single copy and identified as prokaryotic sequences. Inserted viral sequences were not found.

## Zusammenfassung

Die Inkubation des aminosäuredefizienten *Escherichia-coli*-AB1157-Bakteriums mit aus einem oligotrophen Gewässer gewonnenen Partikeln bewies das Vorhandensein eines horizontalen Gentransfers (HGT) durch Herstellung aller Funktionen in Revertantenzellen mit Häufigkeiten bis zu 1,94 × 10^−5^. Da hierbei keiner der Marker bevorzugt übertragen wurde, lag offensichtlich eine generalisierte Transduktion vor. Die entdeckten Partikel wiesen strukturelle Analogien mit Vesikeln der äußeren Bakterienmembran (OMV) auf, jedoch enthielten sie große DNA-Mengen. Durch das Phänomen der seriellen Transduktion waren die aus Revertanten stammenden Partikel wiederum infektiös und übertrugen Erbinformation auf defiziente *E.-coli*-AB1157-Zellen. Diese Ergebnisse lassen auf einen neuen Mechanismus des HGT schließen, bei dem mobilisierte DNA in Partikeln von Spender- auf Empfängerzellen übertragen wird. Die extrahierte DNA aus OMV zweier bekannter α‑ und γ‑Proteobakterien (*Ahrensia kielensis* und *Pseudoalteromonas marina*) war >30 kbp lang, wobei alle Sequenzen als prokaryotisch identifiziert wurden und nur als Einzelkopie vorlagen. Eingefügte virale Sequenzen wurden nicht gefunden.

## Introduction

Prokaryotes are unique in reacting to environmental alterations by a fast acquisition of essential and suitable genetic characteristics. Genome comparisons revealed that small 16S rRNA sequence alterations can cause huge differences in the complete gene repertoire. Moreover, even populations of a single 16S rRNA species contain numerous genomic varieties [11].

This genetic flexibility of the prokaryotes is based in an advantage of the horizontal gene transfer among bacteria as well as between bacteria and other organisms [26, 30]. Obviously, the fate of the transferred DNA in the recipient cell relies on the nature of the DNA molecule itself—if it lacks an active vegetative origin, it must be integrated into the host genome for stable inheritance. Genetic exchange, therefore, plays a key role in the evolution of prokaryotes. The mechanisms and vectors responsible for the horizontal DNA transfer include:

1. the uptake of free DNA from the environment, i. e. transformation,
2. the transfer of DNA from a bacterial donor cell to a bacterial recipient cell termed conjugation,
3. the phage-mediated passage of bacterial genes referred to as transduction,
4. gene shuffling by gene transfer agents (GTA), which are unusual bacteriophage-like vehicles of genetic exchange, first discovered in the bacterium *Rhodobacter capsulatus* [33] containing a random 4.5 kb fragment of bacterial genomic DNA [46] that can be transferred between cells [3, 22, 23, 45, 47, 51] and finally
5. the formation of DNA bearing membrane vesicles (MVs), also referred to as “outer membrane vesicles” (OMVs) or membrane blebs.

The latter mentioned mechanism of DNA transfer was postulated in the last decade [20, 24, 35, 38, 40, 52] and was shown to function via formation and shedding of membrane vesicles during growth of Gram-negative bacteria, whereby these membrane vesicles are transporters of genetic information between strains. Remarkably, the phenomenon of generation and detaching of membrane vesicle in Gram-negative bacterial cells is well documented and frequent. Recently [25], the formation of similar vesicles was also recorded for Gram-positive bacteria.

Currently, the interest in OMVs concentrates on the following topics:

- trafficking of cell-cell signals [17], i. e. interspecies communication leading to quorum sensing reaction within the microbial community [32, 34, 41];
- traffic vehicles for the delivery of toxins [16, 21, 36];
- transfer of antibiotic resistance determinants [8, 50];
- utilization as diagnostic tools by clinicians [13];
- delivery of antimicrobial substances [1, 2, 28, 29];
- contribution to innate bacterial defence by absorption of antimicrobial peptides and bacteriophages [31];
- contribution to the dynamics of biofilm formation [43, 44];
- inter-kingdom communication [12, 27, 39].

Another line of interest, which was less emphasized within the frame of the ongoing research, dealt with the transfer of genetic information, namely, uptake of free DNA by bacterial cells via transformasomes [9, 18, 19] and encapsulation of exogenous DNA by outer membrane vesicles [40] as well as DNA transfer within and between genera [15, 20, 40, 52]. Currently, the magnitude of the OMV driven DNA flux has been rather small, ranging from 3 to 36 kbp [10].

In the present review of own investigations, we provide proof that transferable DNA may be well above 36 kbp within OMVs, that OMV infected cells produce again infectious particles, and we offer information on the restoration of deficiencies within an amino acid-deficient strain of *E. coli* AB1157.

## Materials and methods

Details on the various methods concerning sample collection, CsCl density equilibrium centrifugation, bacterial cultures, analytical approaches, transmission electron microscopy and experimental design for gene transfer assays are available in Chiura et al., [7], Velimirov and Hagemann, [49], and Hagemann et al., [14].

## Results

During a number of own ultrastructural pilot studies designed to investigate the formation and growth steps of membrane vesicles in transformed *E. coli* populations, the appearance of DNA bearing OMVs was regularly observed [4–6, 48, 49]. Within the frame of this investigation, we detected OMVs that were of similar morphological appearance as the OMVs so far described in the literature [2, 34, 40] but larger in size (usually above 100 nm in diameter) and revealing a number of surprising features: they were able to transfer genes to recipient cells with high gene transfer frequencies (Tables 1 and 2), and subsequently the recipient cells produced new OMVs. The DNA in newly produced OMVs had increased in length (up to 350 kbp) compared to the initially harvested OMVs (40–60 kbp).

**Table 1:** Gene transfer frequency ± standard deviation from OMVs derived from 0.2 µm filtered seawater concentrate to *E.* *coli* AB1157 designated as “reverted” at 4 different MOIs and corresponding control experiments. *n* = 6

	MOI	0.12	1.0	5.1	20	200
Experiment	All amino acids reverted	0	0	1.39 × 10^−7^	1.46 × 10^−5^	1.94 × 10^−5^
	Leu reverted	4.41 ± 3.04 × 10^−6^	3.33 ± 3.22 × 10^−5^	9.04 ± 2.85 × 10^−3^	1.04 ± 1.62 × 10^−3^	7.34 ± 3.04 × 10^−4^
	Pro reverted	5.73 ± 2.82 × 10^−6^	2.22 ± 2.38 × 10^−5^	8.34 ± 2.38 × 10^−3^	7.42 ± 1.12 × 10^−3^	7.15 ± 0.28 × 10^−5^
	His reverted	3.53 ± 0.92 × 10^−6^	7.41 ± 1.76 × 10^−5^	9.04 ± 3.07 × 10^−3^	9.83 ± 3.88 × 10^−3^	7.34 ± 4.13 × 10^−4^
	Arg reverted	3.09 ± 0.88 × 10^−6^	1.48 ± 0.52 × 10^−5^	9.73 ± 1.90 × 10^−3^	6.03 ± 0.92 × 10^−3^	5.83 ± 1.08 × 10^−4^
Control	MVs + UV ± AB1157 MVs aut. + AB1157 MVs aut./untr. ± AB1157	0	0	0	0	0
	Davis buffer + AB1157 or UC supernat. + AB1157	0	0	0	0	0

*MV* membrane vesicle, *UV* irradiation of OMVs with ultraviolet light, *aut.* autoclaved OMVs, *untr.* untreated OMVs, *UC supernat.* ultracentrifugation supernatant

**Table 2:** Gene transfer frequency ± standard deviation from OMVs derived from transductant colonies (F1) to *E.* *coli *AB1157 designated as “reverted” at a MOI of 5.5 and corresponding control experiments

		All amino acids reverted	Leu reverted	Pro reverted	His reverted	Arg reverted
Experiment	Transcolonies	2.0 ± 1.3 × 10^−6^	12.3 ± 18.1 × 10^−6^	8.77 ± 8.74 × 10^−6^	3.16 ± 0.65 × 10^−6^	12.0 ± 1.9 × 10^−6^
Control	MVs + UV ± AB1157 MVs aut. + AB1157 MVs aut./untr. ± AB1157	0	0	0	0	0
	Davis buffer + AB1157 or UC supernat. + AB1157	0	0	0	0	0

*MV* membrane vesicle

**Fig. 1:**
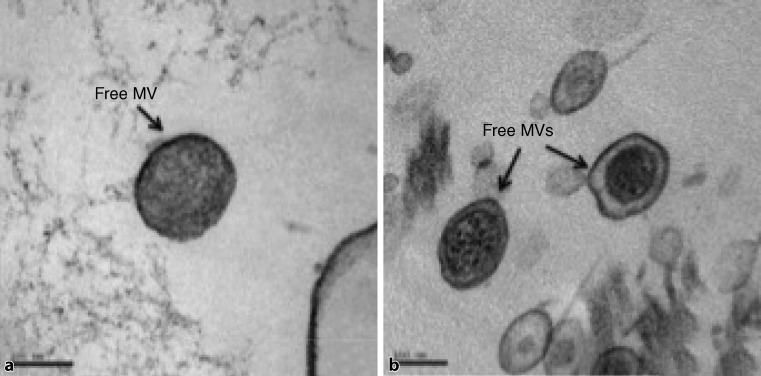
Membrane vesicles (MVs) produced from **a** *Pseudoalteromonas marina* and **b** *Ahrensia kielenis* during the stationary phase

**Fig. 2:**
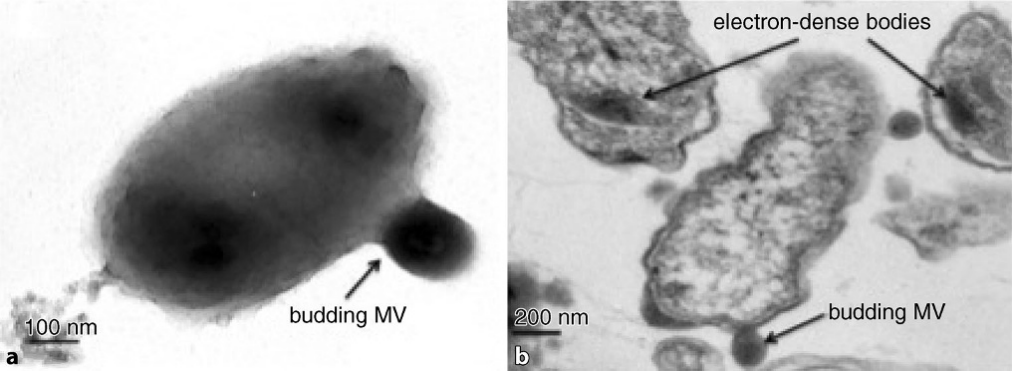
Membrane vesicle (MV) production: **a** Negative stained total view of a bacterial cell in the process of budding. **b** Ultrathin section of bacterial cells in the process of budding

**Fig. 3:**
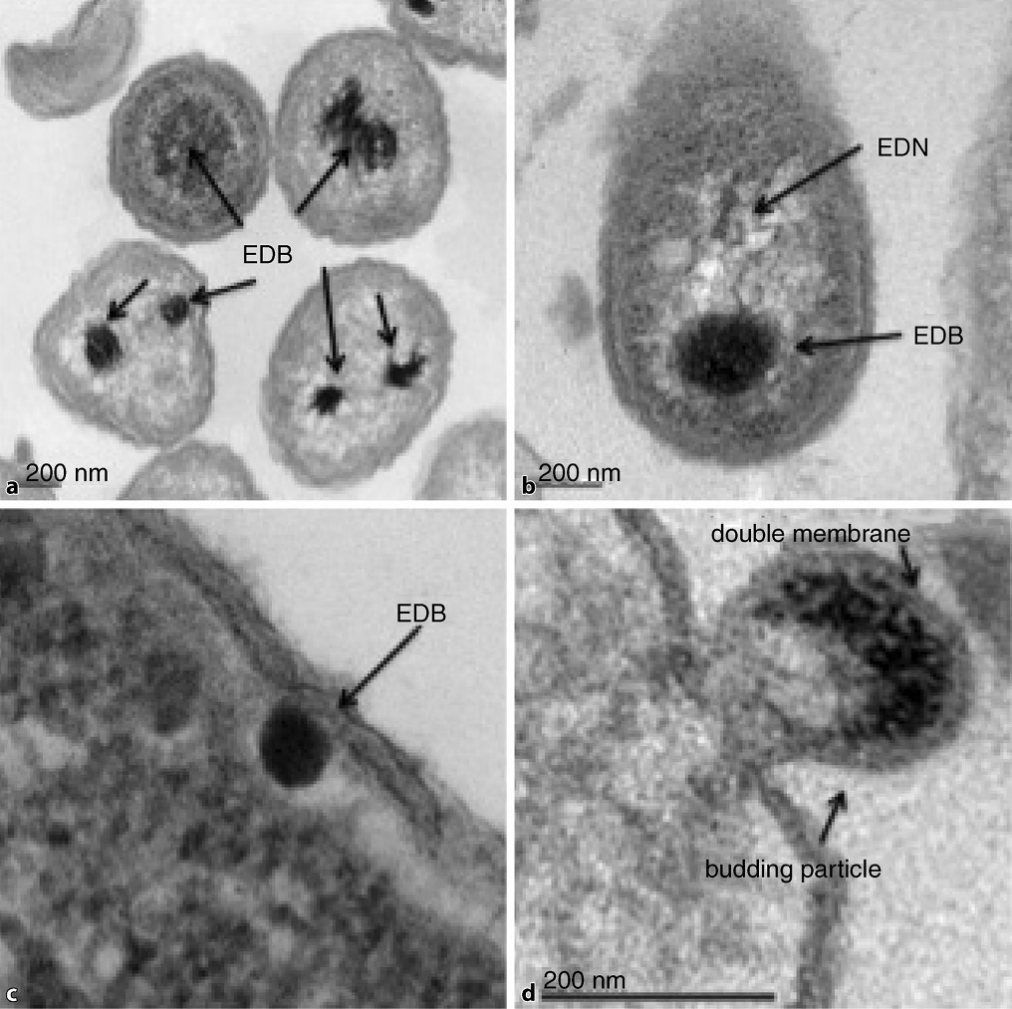
Thin sections of transductants, demonstrating the structure of electron dense bodies (EDBs). **a** EDBs of different electron densities and sizes. **b** Higher magnification of an EDB in the vicinity of an electron dense network (EDN) within the recipient cell. **c** EDB in contact with the inner layer of the cell membrane. **d** Budding structure displaying features of a double membraned vesicle

In contrast, previously published information on DNA containing OMVs indicated DNA lengths ranging from 3 to 36 kbp [10]. In follow-up studies [7, 49] and during preparatory investigations where we inspected over 1000 TEM slides and investigated whether OMVs within the mentioned size fraction (>100 nm in diameter) from natural seawater would trigger the above quoted features in recipient cells, the following traits were recorded for the produced outer membrane vesicles:

1. The observed vesicles appeared already in harvestable quantities before the end of the logarithmic phase of the recipient cell culture but reached their maximum in the stationary phase (Fig. 1).
2. The investigated particles were DNA carrying OMVs.
3. The packaged DNA ranged from 50 to 80 kbp.
4. First sequencing data pointed out that the vesicle DNA consisted of bacterial DNA.
5. The number of visible MVs (burst size) within the cells was low, ranging between 1 and 5 per bacterial cell.
6. The OMVs were released by budding (Fig. 2).
7. The observed vesicles infected other bacterial cells and repaired genetic deficiencies (Table 1). It could be repeatedly shown that the incubation of cells belonging to the amino acid-deficient strain of *E. coli* AB1157 with membrane vesicles revealed evidence of HGT by restoration of all deficiencies (markers) in revertant cells with frequencies between 1.39 × 10^−7^ for a multiplicity of infection (MOI) of 5.1 to 1.46 × 10^−5^ for a MOI of 20. The highest gene transfer frequency was obtained for single markers with values up to 1.04 × 10^−2^ at a MOI of 20. These obtained gene transfer frequencies belong to the highest reported frequencies and only the values recorded by McDaniel [37] were higher than those obtained from our investigations.
8. This horizontal gene transfer between species has all characteristics of a generalized transduction [42] as none of the markers was preferentially transferred.
9. Obtained transductants were able to produce new infective vesicles that were again released via budding. These vesicles were of larger size than the primary infecting vesicles. The observed process of consecutive infection was termed serial transduction and the DNA of the resulting OMVs can reach a length of about 350 kbp.
10. Investigations with the transmission electron microscope (TEM) of experimentally infected *E. coli* cells revealed the appearance of distinct electron dense structures and bodies (EDB), which had never been observed until now (Fig. 3a,b,c), considered as precursors of budding membrane vesicles (Fig. 3d).
11. The OMVs, mostly derived from alpha-proteobacteria, were also able to infect phylogenetically distant bacterial species such as gamma-proteobacteria (*E. coli* AB1157) and induce intergeneric transduction, thus functioning as gene mediators for a broad host range.

Using TEM in a different approach, inspected OMVs from the alpha-proteobacterium *Ahrensia kielensis* and from *Pseudoalteromonas marina*, a gamma-proteobacterium, revealed two kinds of vesicular bodies: a bilayered form of OMVs with diameters between 30 and 250 nm, but also OMVs exhibiting double bilayers and diameters ranging between 80 and 200 nm. While the bilayered OMVs could be distinguished either by a large electron-dense structure or were electron translucent, the double bilayered ones showed the electron dense substance in contrast in the core region within the intermembrane space of the first and second bilayer (Figs. 1 and 3).

Furthermore, 30,094 bp of the genome from OMVs of *A. kielensis *and 45,981 bp of *P. marina* were sequenced. The findings pointed out that the sequences existed only in single copy and, except for one, had prokaryotic equivalences. Inserted viral sequences were not detected. We found no hint in the OMVs under investigation for any OMV-specific genomes. Some of the analyzed sequences code for proteins which are membrane associated like extracellular solute-binding protein family 3, TonB family protein, TonB-dependent receptor protein, and ABC transporter permease. It should be noted that proteins involved in defence and survival strategy (e. g. the putative TetR family transcriptional regulator or the toxin–antitoxin system, toxin component, RelE family) were detected.

## Discussion

Although the OMV–DNA complex is a subject of great interest and hence profoundly under investigation, unexpectedly less is known about sequence information. In this short review we show that our investigation [14] was the first to report that the DNA incorporated in bacterial OMVs contains a large spectrum of protein coding sequences. These DNA sequences are either encapsulated in regular bilayered membrane vesicles which originate from the outer membrane of the bacterium or in the more difficult design of the OMV double bilayer types consisting of a further inner membrane layer being probably derived from the cytoplasmic cell membrane. Electron dense structures (EDS) analog to those shown in thin sections (Figs. 2 and 3) were also monitored in prior studies of revertant E. coli strains in liquid culture during OMV production [7]. In these earlier investigations, gold-labelled anti-DNA antibodies bound to likewise electron dense structures were used, which we named electron dense bodies (EDBs), thereby demonstrating the presence of DNA in these EDBs. Despite our finding of a clear analogy in our TEM slides to our previous results, we decided to refrain from presuming that DNA is necessarily a fraction or a part of the EDBs in thin sections of *A. kielensis* or *P. marina* while attempting to find precursor structures for the OMVs. As bacterial DNA fibers are often but not always associated with polyphosphate bodies, which have a similar electron dense appearance as the observed EDBs, we could not verify that all the detected EDBs were precursor structures for the DNA to be encapsulated in OMVs prior to budding. Nonetheless, it was assumed that the majority of these structures were precursors as a distinct granulation in the DNA carrying EDBs was regularly observed, which was absent in polyphosphate bodies.

The described process of horizontal gene transfer via OMVs leads to a number of assumptions, which need to be mentioned. The finding of restored functions of all markers in revertant cells at MOIs of 5, 20, and 200, demonstrates that multiple infections may have taken place. At the present state of knowledge, one can only presume the possible effects of the noted gene transfer strategy on bacterial populations. Burst size, which can be equated with budding size, indicates that the abundance of OMVs may well be below that of surrounding bacteriophages. Nonetheless, all our investigations suggest that OMVs are by far more efficient in transferring genes than bacteriophages.

A surprising feature of the investigations was that the result of this HGT are revertants, which produce themselves anew infectious OMVs with increased DNA length, reaching a DNA content of >350 kbp.

All so far listed features of the investigated OMVs, namely release via budding, particle-related transfer of large amounts of DNA to recipient cells and their potential for serial gene transfer, show that we observed an important and new mechanism for HGT between prokaryotic cells. Furthermore, the production of OMVs may be a strategy to ensure a back-up of genetic information in case of nutrient shortage leading to starvation of bacterial populations. Assuming that transfected bacterial cells produce some 350 kbp per OMV in the stationary phase and that each particle carries a unique piece of the *E. coli* chromosome, then 13–20 of such OMVs would be sufficient to save the entire bacterial chromosome. To which extent the knowledge about the functions of OMVs and associated DNA may be used for medical applications, e. g. to support DNA repair in preventing carcinogenesis, remains a matter of debate and experimentation in the future.
